# Effect of Cannabidiol on the Long-Term Toxicity and Lifespan in the Preclinical Model *Caenorhabditis elegans*

**DOI:** 10.1089/can.2020.0103

**Published:** 2021-12-09

**Authors:** M. Hunter Land, Marton L. Toth, Laura MacNair, Siva A. Vanapalli, Timothy W. Lefever, Erica N. Peters, Marcel O. Bonn-Miller

**Affiliations:** ^1^ Canopy Growth Corporation, Smiths Falls, Ontario, Canada.; ^2^ NemaLife, Inc., Lubbock, Texas, USA.; ^3^ Texas Tech University, Lubbock, Texas, USA.

**Keywords:** cannabidiol, toxicity, lifespan, motility, aging, health

## Abstract

**Introduction:** Despite widespread use of cannabidiol (CBD), no lifelong toxicity study has been published to date. *Caenorhabditis elegans* is often used in preclinical lifelong toxicity studies, due to an estimated 60–80% of their genes having a human ortholog, and their short lifespan of ∼2–3 weeks. In this study, we examined both acute and long-term exposure studies of CBD at physiologically relevant concentrations.

**Materials and Methods:** Acute toxicity was determined by treating day 1 adults with a wide range of CBD concentrations (0.4 μM to 4 mM) and assessing mortality and motility compared to control animals. Thermotolerance was examined by treating adult animals with CBD (0.4 μM to 4 mM) and exposing them to 37°C for 4 h, and then scoring for the number of alive animals treated with CBD compared to controls. Long-term toxicity was assessed by exposing day 1 adults to 10, 40, and 100 μM CBD until all animals perished. Control animals had no active drug exposure.

**Results:** We report both acute and long-term exposure studies of CBD to adult *C. elegans* at physiologically relevant concentrations. Acute toxicity results showed that no animal died when exposed to 0.4–4000 μM CBD. The thermotolerance study showed that 40 μM CBD, but not other treatment levels, significantly increased resistance to heat stress by 141% compared to the untreated controls. Notably, whole-life exposure of *C. elegans* to 10–100 μM CBD revealed a maximum life extension of 18% observed at 40 μM CBD. In addition, motility analysis of the same groups revealed an increase in late-stage life activity by up to 206% compared to controls.

**Conclusion:** These results serve as the only CBD lifelong exposure data in an *in vivo* model to date. While further research into the lifelong use of CBD should be carried out in mammalian models, the *C. elegans* model indicates a lack of long-term toxicity at physiologically relevant concentrations.

## Introduction

Over the last decade, extensive research has shown that cannabidiol (CBD) has the potential to treat a variety of disorders. Extensive preclinical work examining the ability of CBD to reduce epileptic seizures began in 2007, resulting in expanded knowledge of CBD pharmacology and identification of new disease targets. Studies have highlighted the therapeutic potential of CBD to treat conditions ranging from epilepsy and autism, to anxiety and psychiatric disorders.^1^ Dosing and duration of use have varied extensively across studied indications, with some demonstrating clinical efficacy with single acute doses of 150 mg and others requiring over 1500 mg daily to demonstrate benefit.^1^

A major hurdle in the acceptance of CBD as treatment for chronic conditions relates to the general paucity of long-term safety data. From 2013 to 2018, there were over 1800 patient-years of data generated by the Epidiolex^®^ clinical trial programs.^2^ These data revealed some safety concerns following repeated administration of high doses (e.g., 20 mg/kg).^2,3^ While these observations are an important initial step, long-term CBD safety data are still missing from the literature. Indeed, the U.S. Food and Drug Administration (FDA) has publicly stated that data gaps on long-term safety risks hinder the establishment of guidelines for the safe and legal use of CBD in foods and supplements. Given that data on the long-term CBD use in humans and mammalian surrogate models may not be available for some time, a well-established preclinical model of long-term toxicity is critical to immediately inform evidence-based regulation of CBD.

The nematode *Caenorhabditis elegans* is one species that has gained widespread acceptance in preclinical models of lifelong toxicity associated with drug exposure, due to their transparency at all stages of life, their short lifespan (2–3 weeks), and an estimated 60–80% of its genes having a human ortholog.^4^ *C. elegans* has intact and metabolically active digestive, reproductive, endocrine, sensory, and neuromuscular systems and, in previous studies, *C. elegans* toxicity ranking assays have been shown to be comparable to rodent models in reflecting the effect of drugs on the whole organism.^5^ Motility in *C. elegans* involves coordinated neuromuscular actuation to generate propulsive thrust for animals to crawl. Drug-induced declines in motility inform an organ-level toxicity compared to death, which is indicative of whole-organism toxicity and physiology.^6^ Because mortality and motility can easily be measured, *C. elegans* is frequently used to screen drugs for toxicity and efficacy, especially in the areas of neurodegenerative disease and aging (e.g., metformin, resveratrol, and DAF-16/FoxO transcription factor).^7,8^ These studies demonstrated life and health span extension outcomes paralleling those in mammalian systems.^9^

The objective of this study was to examine the effects of CBD on acute and lifelong toxicity, lifespan, and aging as measured by mortality and motility in *C. elegans*. This study addresses critical data gaps in long-term CBD toxicity, and helps inform the FDAs evolving CBD regulatory framework.

## Materials and Methods

### Culturing, age synchronization, and microfluidic chip loading

Wild-type Bristol (N2) *C. elegans* (*n*=3504) was used in this study (Caenorhabditis Genetics Center, MN). For age synchronization, animals were cultured on nematode growth media (NGM) plates (60 mm Petri dishes; Fisher Scientific) on a standard food source of 20 mg/mL *Escherichia coli* OP50 and incubated for 48 h at 20°C. Gravid adults were loaded into microfluidic chips^10^ (Infinity Chips; NemaLife, Inc., TX) and allowed to lay eggs for 2 h (coded as day 0). The egg suspension from the chips was flushed to fresh NGM plates and incubated at 20°C. Four days later, young adult animals were loaded into microfluidic chips along with 20 mg/mL of *E. coli* OP50 in liquid NGM with 0.2% dimethyl sulfoxide (DMSO).

Before use, microfluidic chip interiors were filled with 70% ethanol (Fisher Scientific) for 5 min. Subsequently, the device was rinsed four to five times with NGM solution and treated with 5 wt% Pluronic F127 (Sigma-Aldrich) for 30 min. The Pluronic-treated devices were stored in moist Petri dishes at 20°C for immediate use and at 4°C for future use.

### Formulation

Several concentrations of hemp-derived CBD (99.81% purity; Canopy Growth Corporation) were formulated in liquid NGM and mixed with 0.2% DMSO (Fisher Scientific). A wide range of CBD concentrations was tested in the acute toxicity and thermotolerance assays: 0.4 μM, 4 μM, 10 μM, 40 μM, 100 μM, 400 μM, and 4 mM, spanning subphysiologic, supraphysiologic, and physiologic concentrations demonstrated in clinical trials, assuming homogeneous distribution.^11^ In all tested solutions (those containing CBD and not), the final concentration of DMSO was maintained at 0.2% v/v and the food concentration was maintained at 20 mg/mL of *E. coli* OP50.

### Toxicity assays

For acute toxicity assays, day 1 adults were loaded into microfluidic chips along with 20 mg/mL of *E. coli* OP50 and 0.2% DMSO NGM solution alone (control) or with CBD concentrations described above (0.4 μM to 4 mM). Videos of animal population in the chips were recorded and scored using the Infinity Screening System (NemaLife, Inc.) at three time points: 0, 3, and 6 h.

The degree to which animals resist heat stress (thermotolerance) has been frequently used to predict the long-term effect of compounds,^12^ suggesting that declines in thermotolerance inform understanding of long-term toxicity. For thermotolerance assays, day 1 adults were loaded into microfluidic chips for 48 h at 20°C with 0.2% DMSO NGM solution alone (control) or CBD concentrations ranging from 0.4 μM to 4 mM. Subsequently, animals were exposed to 37°C for 4 h, followed by 30 min of recovery and scoring for the number of alive animals in the chips by visual inspection of animal movement.

For lifelong toxicity assay, concentrations consistent with physiologically relevant CBD doses utilized in human clinical trials were selected for lifelong toxicity assays (10, 40, and 100 μM).^11^ The lifelong assay was initiated by loading day 1 adults into the microfluidic chips. Fresh DMSO (control) or CBD solutions were administered daily, and chips were incubated at 20°C until all animals perished. Videos were acquired each day before and after feeding fresh DMSO or CBD solutions to determine live counts and animal motility. Control animals had no active drug exposure, only 0.2% DMSO.

Each *C. elegans* assay was conducted in triplicate, except for the acute toxicity assay, and each replicate consisted of two or more technical replicates. One technical replicate is a population in a microfluidic chamber (with >60 animals for chronic and >15 animals for acute assays).

### Statistical analyses

The acute and lifelong toxicity assay videos were analyzed using the Infinity Code software (NemaLife, Inc.) for animal survival and motility. The number of living animals in the population was determined based on detectable movement. Motility was determined based on the displacement of individual animals from the rectangular area (bounding box) that encloses their whole body. Animals that moved more than their body length within 30 sec were labeled “highly active.” The percentage of highly active animals in the population was then calculated.

One-way ANOVA was used to compare the nonexposed control and CBD-treated animals in the acute and thermotolerance assays. Kaplan-Meier curves from the lifelong toxicity assay were generated using GraphPad Prism [Version 8.4.2 (679)]. Log-rank test was used to compare the survival curves between the nonexposed control and CBD-treated populations. We also utilized a multiple comparison test, using the Bonferroni method, to determine the concentrations that provided significant thermotolerance benefit.

## Results

**FIG. 1.**
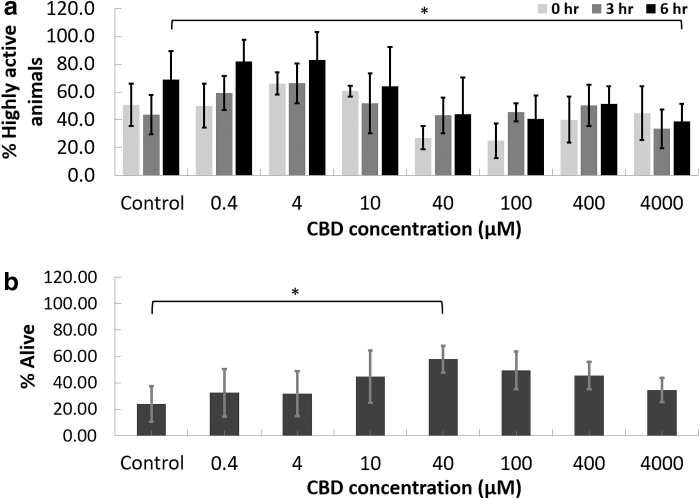
Acute toxicity and thermotolerance response of CBD on *Caenorhabditis elegans*. **(a)** Percent highly active animals of *C. elegans* at 0, 3, and 6 h after exposure to CBD (*n*=53–65 animals per concentration). **(b)** Percentage of *C. elegans* alive after 30 min recovery from heat stress (4 h at 37°C) at various concentrations of CBD (*n*=156–203 animals per concentration). Data are mean±standard deviation. *p<0.05 indicates a statistically significant difference between CBD-treated and control samples. CBD, cannabidiol.

The acute toxicity results showed that no animal died prematurely under any of the tested CBD concentrations (data not shown), and only 4000 μM CBD had a significant negative effect on motility. Highly active animals declined from 69% at 0 h to 39% at 6 h at 4000 μM (*p*<0.05; Fig. 1a). Due to the absence of any death event, or other signs of toxicity, only one replicate was performed. An LC_50_ was not identified.

Results from the thermotolerance assay showed that 40 μM CBD treatment significantly increased resistance to heat-induced molecular stress by 141% compared to the untreated controls (Fig. 1b). While a trend toward increased thermotolerance was seen at 10 and 100 μM, increases did not meet statistical significance.

**FIG. 2.**
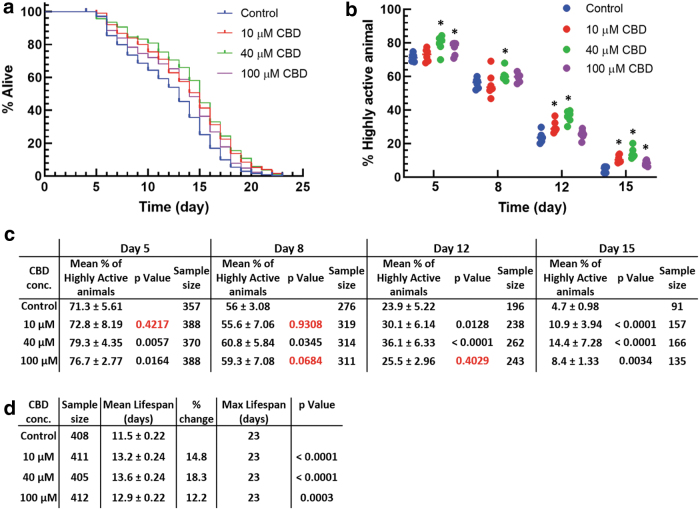
Effect of long-term exposure of CBD on *Caenorhabditis elegans* survival and locomotor activity. **(a)** Survival curves at 10, 40, and 100 μM CBD. Survival data shown are pooled from three biological replicates with each biological replicate having two technical replicates. **(b)** For the same population in **(a)**, percentage of highly active animals is shown at four time points in life. Mean and *p*-values, sample size for motility plots, and survival curves are shown in tables **(c, d)**, respectively.

Lifelong exposure to CBD did not have significant negative effects on lifespan or motility (Fig. 2). Extension in mean lifespan compared to controls was observed for 10, 40, and 100 μM CBD, significantly extending *mean* lifespan by 14.8%, 18.3%, and 12.2%, respectively (*p*<0.0001, *p*<0.0001, and *p*<0.001, respectively, Fig. 2a, d). No significant changes to *maximum* lifespan were observed; control and treated populations all lived up to 23 days. In addition, 40 μM CBD treatment increased the percentage of highly active animals at all stages of life, including old age, compared to controls (Fig. 2b, c). On days 5, 8, 12, and 15 at 40 μM CBD, the percentage of highly active animals increased by 11.2%, 8.6%, 51.0%, and 206.4%, respectively, compared to controls (days 5, 8, 12, and 15; *p*<0.01, *p*<0.05, *p*<0.0001, and *p*<0.0001, respectively). Concentrations of 10 and 100 μM CBD also significantly increased the percentage of highly active animals; however, at a lower rate and only on certain days (10 μM, days 12 and 15, 25.9% and 131.9%, *p*<0.05 and *p*<0.0001, respectively; 100 μM, days 5 and 15, 7.6% and 78.7%, *p*<0.05 and *p*<0.0001, respectively).

## Discussion

CBD did not demonstrate any degree of acute or lifelong toxicity or related liabilities at physiologically relevant concentrations. Indeed, CBD extended mean lifespan up to 18.3% and increased late-stage life activity by up to 206.4% compared to controls. At low (10 μM) and medium (40 μM) concentrations, CBD attenuated age-related declines in motility on days 12 and 15. Moreover, treatment with 40 μM CBD significantly increased thermotolerance compared to the untreated controls. Acute toxicity assays with CBD did not have a significant impact on *C. elegans* survival or motility, with the exception of 4000 μM CBD, which significantly reduced motility; however, this concentration is at least 10 times outside of physiologically relevant concentrations.

The U.S. regulatory implications of the findings demonstrated by this study are of paramount importance. Following the passage of the Agriculture Improvement Act of 2018,^13^ also known as the “Farm Bill,” hemp-derived CBD was removed from the Controlled Substance Act. Under this regulatory framework, management of CBD product manufacturing and sale falls under the FDA and Federal Trade Commission, yet no formal regulations of manufacturing or sale have been implemented. Widespread use of CBD products continues to proliferate, despite lack of regulation. The FDA has publicly stated that two significant concerns that are both difficult to address and have little available data remain a barrier to formalized regulations: safe levels of CBD for daily consumption and the effects of lifelong use.^14^ The results of this study directly address these two needs, and fill some of the data gaps needed to establish evidence-based safety-oriented regulations.

Because this study was the first to examine the effects of lifelong exposure to CBD, many unanswered questions remain. First, although CBD extended lifespan and increased measures of health span in this *C. elegans* model, future research is needed to determine potential mechanisms for these findings. Similar to metformin,^15^ CBD can inhibit folate and methionine metabolism,^16^ suggesting at least one mechanism for future exploration. Second, data from this study can only be used to inform our understanding of toxicity associated with CBD isolate. These data do not inform toxicity associated with more complex botanical CBD formulations (e.g., “full spectrum” and “broad spectrum”), or the over 120 other potentially biologically active phytocannabinoids produced by the cannabis plant.^17^ Similar studies should be conducted on other cannabinoids in isolation and combination, given widespread commercial availability of cannabinoid formulations. Finally, this study was conducted with wild-type Bristol (N2) *C. elegans.* While this strain of *C. elegans* is most commonly used in lifelong toxicity studies,^5^ findings suggest that future research would benefit from examining the studied effects in strains known to suffer from deficits in motility and thermotolerance to determine whether CBD could act as a potential novel therapeutic agent for prevention of age-related decline in motility and improvement in longevity.

In conclusion, CBD extended lifespan and drastically improved activity levels in the late stage of life at studied concentrations of 10, 40, and 100 μM. Furthermore, no toxicity effect was observed at any concentration tested (0.4 μM to 4 mM) in any study, outside of the decrease in motility observed at 4000 μM. These findings address critical gaps associated with lifelong toxicity of CBD, and can inform FDA regulation surrounding CBD safety.

## Abbreviations Used

CBD: cannabidiol
DMSO: dimethyl sulfoxide
FDA: Food and Drug Administration
NGM: nematode growth media
